# Exploring Primary Care Streaming Pathway in Emergency Departments in Saudi Arabia: A Qualitative Study

**DOI:** 10.1155/2023/7045983

**Published:** 2023-04-21

**Authors:** Marwah Hawsawi, Bayan Alilyyani

**Affiliations:** ^1^Nursing Department, Ajyad Hospital, Ministry of Health, Makkah Al Mukarramah, P.O. Box 24331, Saudi Arabia; ^2^Nursing Department, College of Applied Medical Sciences, Taif University, P.O. Box 11099, Taif 21944, Saudi Arabia

## Abstract

**Background:**

Due to significant emergency department overcrowding, some hospitals implemented a system of directing certain patients who were deemed not in need of emergency care to other facilities called triage away. Pathways were developed as ways to stream patient from emergency departments to primary healthcare who is presenting with less urgent or nonurgent conditions. Thus, the purpose of this study was to explore the pathways (process) for streaming patients from emergency department to primary healthcare at three different sites across Western Region of Saudi Arabia and to identify the streaming criteria and guidance.

**Materials and Methods:**

This study used a qualitative observational design. Data were collected through an unstructured observational approach, with an in-depth case study observation involving three emergency departments in the Western Region. Data were collected over three months until data saturated and recorded in the form of filed notes.

**Results:**

The results of this study explored that all CTAS-5 were streamed away either (*off-site*) or (*on-site*) from emergency department. The average of the sorting/triage cases were around 200 to 250 per shift, and about third to half of them were streamed to Primary Health Centre or Urgent Care Clinic. The total streamed patients were ranging from 50 to 60 per shift, which mean 15–20 case per hour. The study highlighted many factors that influence the practice and decision of streaming.

**Conclusions:**

In general, the term “streaming” was not as widely known among emergency clinicians, as was the term “triage.” However, streaming was performed as an evidence-based practice, and clinicians routinely acted to direct patients based on hospital policies. Although, in one hospital, some nurses hack the system to manage the flow of patients based on their intuition. In contrast, the nurses in another hospital emphasised the importance of experience and confidence in streaming improvement.

## 1. Introduction

The high turnout of patients to the emergency department is overburdening healthcare providers and the systems which put them at risk because of overcrowding [1–4]. Managing outflow to the emergency department is the main goal to maintain the high quality of care and to ensure patient safety and satisfaction [5]. The scope of care provided in the emergency department varies depending on the patient's condition and severity that ranged from immediate to minimal or nonurgent. Moreover, the triage and reception area are the front line that faces the stream paths and the indicative roadmap for patient care, which plays an important role in achieving the main objective. For instance, they are directing the patient to the appropriate area or facility either in or next to the emergency department to meet their needs. Approximately, 15–40% of all cases treated in the emergency departments can be managed and treated in primary healthcare [6].

In Saudi Arabia, it is not far from international estimates; around 42.2% of the patients who attended emergency departments (EDs) classify as level-V (nonurgent) depending on the Canadian Triage and Acuity Scale (CTAS) guidelines; however, 16.8% of patients were redirect to Primary Health Centre (PHC) [7]. There are different reasons for nonurgent patients for visiting EDs in Saudi hospitals including lack of services and healthcare providers in primary care, availability of services in EDs and fast access for all patients, and patients' thoughts regarding to receive the best care in EDs [8]. These factors affect the rate of visiting EDs which leads to overcrowding. Moreover, they may cause delays in providing the best quality of care for patients. As a result, increasing waiting time and overcrowding in EDs and decreasing the quality of care for patients who really need to be treated in EDs are major consequences of attending EDs for nonurgent patients [9]. Consequently, applying the streaming pathways will simplify patient service and improve the overall outcomes.

Pathways were developed as ways to stream the patient from the emergency department to Primary Care Centres who is presenting with less urgent or nonurgent conditions, depending on streaming or triage criteria, which includes streaming, triage, primary care clinicians selecting their own patients, and redirection [10]. Thus, pathways are designed to facilitate and expedite services and meet the patient needs in an efficient and safe manner. However, streaming or redirecting patients must be applied under certain circumstances and criteria and carried out by certified, qualified, highly educated, and well-trained healthcare providers [11]. Therefore, the streamer must ensure the patient's right to health (availability, accessibility, acceptability, and quality), as well as satisfaction with the provided care, which are introduced and articulated as essential elements of the right to health see (Table 1) [12, 13].

Streaming to primary care is marked by significant policy and standards established in 2017, to promote patient service and safety [11, 14]. On the other hand, streaming practices could be suitable or unsuitable to the patient affected with some factors. For example, some general practitioners/streamers fail to communicate and gain patient trust resulting in unsuitable streaming, which is recognised as lower grade than the actual with acute signs by sending the patients to PHC at the moment they need emergency intervention. Furthermore, redirecting patients who are suitable for PHC to ED could lead to misuse the resources from both sides (ED and PHC) as another form of unsuitable streaming. Thus, when the benefit and satisfaction with the service provided are achieved, it is called suitable streaming [15]. To exclude such events, some knowledge and skills must be acquired to regulate patient flow to ED by triage or streaming nurse such as triage, simple assessment, and redirection [5, 14, 16, 17] (See Table 2).

Thus, the goal of this study was to explore the pathways (process) for streaming patients from emergency department to primary healthcare at three sites across Western Region in Saudi Arabia.

## 2. Materials and Methods

### 2.1. Design

The qualitative observational design was used to achieve the goal of this study.

### 2.2. Setting

This study was conducted in emergency words in three different sites in Western Region, Saudi Arabia. The three sites were selected purposively to be similar in triage system, ED levels, and organisational structure. It was performed in three cities which were Makkah (Ma), Taif (Ta), and Jeddah (Ja). All three hospitals are operated by Saudi Ministry of Health.

### 2.3. Sample Size and Selection Approach

All cases streamed away from the emergency department by certified healthcare providers who cover triage area, as well as formal non-healthcare workers who account for the front door such as receptionist. The sampling target three emergency departments with same models of care and triage system with or without primary care services models. Visit to each study area were scheduled and arranged with hospital managers. However, uncertified/unlicensed healthcare providers such as paramedic and informal non-healthcare workers such as security were excluded.

A continuous flow of cases was observed in both sites Ma and Ja hospitals, while an intermittent flow was observed in (Ta), both in the morning and evening shifts as well as on weekends. Assembling the sample were started with unstructured interview with the Chief Director of ED or his representative, for permission and overview of their ED system and structure, as well as their policy and procedures, for all the three sites. In addition to the Nursing Directors, followed by open conversation with the triage Doctor and Nurses to answer research questions. Actually, the total number was 5–7 doctors and 10–15 nurses for each study site. However, the total average of the sorting/triage cases were around 200 to 250 per shift and about third to half of them were streamed to Primary Health Centre or Urgent Care Centre (PHC/UCC).

### 2.4. Data Collection Plan

Unstructured observational approach was used to collect study data involving watching and recording behaviours and patterns of streaming and redirecting patients without participating in activities. In-depth “case study” observation includes three emergency departments with different sizes, flow rate, and geographical locations throughout Western Region. The visits were scheduled after obtaining ethical approval between August and November 2022. Visiting each case study was between 5 and 7 days. However, observation was carried out within working hours including weekdays and weekends.

The data were collected from unstructured observation recorded in form of filed notes supplemented with information from unstructured interviews or conversation with stakeholders. For example, “key informants may be asked to describe what went on time that the observer was unable to attend or to describe events that occurred before entering the filed” [18]. Moreover, to manage collected data, each entry should have the date, time, and location the observation was made.

### 2.5. Analysis

**Figure 1:**
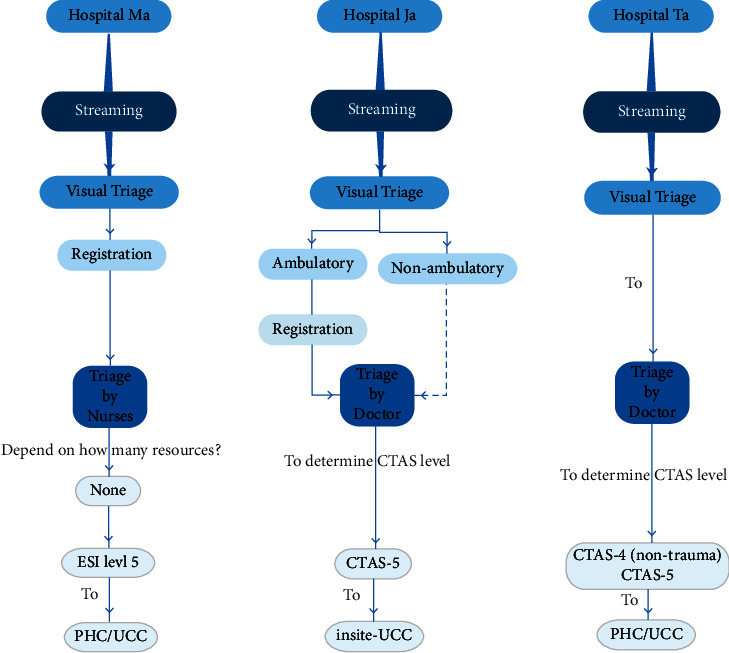
Streaming pathways in the emergency department.

All collected data (field notes, information from unstructured interview, and conversations) were analysed using Lininger's unhousing method to illustrate the paths used in field setting, which involves four-phase sequence “collect, describe, and record data; identifying and categorising descriptors; analysing data to discover repetitive patterns; and abstracting major themes and presenting finding” [18]. Furthermore, thematic analysis was one of the ways to understand qualitative data analysis. Field notes from case study visits were analysed to explore themes related to primary care streaming. However, produced themes from observation at case study sites used to create a set of draft pathways for methods of streaming (See Figure 1).

#### 2.5.1. Rigour in Qualitative Research

To achieve rigour in our qualitative study by applying the unique components (credibility, transferability, dependability, and confirmability). In order to establish authenticity, a nonparticipant observer (PI) spent as much time in the sites as a complete observation in addition to using the interviewers' words and unstructured conversations. On the other hand, we used the same data collection methods and sheets with the three different geographical locations (study sites), to achieve transferability. In term of dependability, we followed the step-by-step unhousing Lininger method to provide a detailed description of the analysis of the research data and to identify similarities in the results. As a result of establishing the credibility, transferability, and dependability we have been achieving the confirmability by documenting the field notes from case study visits.

### 2.6. Ethical Approval

Ethical approval for case study visits was obtained from Ministry of Health-Research Ethics Committee. IRB Number: H-02-K-076-0722-779, Date of Issue 15.08.2022.

## 3. Results

Streaming and triage assessment were both used in the three study sites in the emergency departments, either at front door or inside the ED, by clinicians (physicians or nurses) depending on hospital policy. One of the three locations do not apply streaming (triage away). However, the three hospitals unanimously agreed to stream level-5 cases based on the hospital policies, although the triage system is different. Furthermore, Ma and Ta hospitals are taking (off-site) model of care, while a Ja Hospital (on-site) model of care. On the other hand, the average number of patients streaming to PHC/UCC (Primary Health Centre/Urgent Care Centre) during weekdays and weekends was between 30 and 40 cases in the morning and between 50 and 60 cases in the evening, at the three study sites. Thus, the most common cases were medically free and vitally stable. For example, old trauma or fall-down, sore throat, flank and abdominal pain, COVID-19 signs without fever, and dental pain and dressing. To illustrate, *off-site* means away from the hospital site to other healthcare facilities for either some specialised services such as gynaecology or primary care services while *on-site* means within the hospital site or parallel at front door to emergency department.

The three main themes to explore the streaming practice include streaming methods/protocol, criteria and experience of the ED clinician, and streaming decisions and outcomes.

### 3.1. Streaming Methods and Protocols

An *on-site* model of care is applied at hospital (Ja), as part of hospital care, located several steps away from the ED, on the opposite side. Streamed patients away from ED either to PHC during working hours or to UCC outside working hours based on Emergency Severity Index (ESI) and the number of resources the patient needs.

However, all patients presenting to the ED at (Ma) hospital are screened, assessed, and prioritised to identify their healthcare needs. Therefore, the assessment includes vital signs and complete history of the current chief's complaint, often performed by senior ED nurses about 90%, unless if the patient was elderly or hysterical, the physician carried out the streaming. Moreover, the triage nurse asked the patient about his/her chief complaint, perform pain assessment, take the vital signs and accordingly categorise the patient based on the ESI system and print him/her triage slip number then direct him/her to waiting area and enter the cubicle once her number is called.

ESI-5 patients were triage and direct either to PHC or UCC, (*off-side*) model of care. Figure 1 shows the paths where the patients are first seen by visual triage nurse at the front door to identify respiratory cases then directed to registration desk to complete the patient data and will then be waited for triage. On the other hand, at (Ta) hospital, all patients presenting to ED are prioritised based on the established triage criteria. Triage assessment (brief assessment) will be performed by a qualified/competent triage doctor within 5 minutes upon arrival of a patient. The features to be used to assess urgency are combination of the presenting problem and general appearance of the patient, possibly combined with physiological observations. Based on the results of screening, Patients will be accepted only if the organisation can provide the necessary services and the appropriate inpatient care setting, CTAS-4 and -5 should be instructed by the visual triage physician to avail the services of Primary Health Centres.

Therefore, the triage nurse fills out all items (demographic data, vital signs, and triage No) in the electronic triage form for all CTAS categories in the triage area. Then, print the form and handover to the patient or relative. Thus, the triage nurse will advise the patient and direct them to the nearest centre. In addition, the active UCC list was printed and posted on the wall in sight of the patients. Totally, 9 centres have been distributed, one for each sector, working 18 hours a day, 7 days a week, in two Shifts, in (Ma) hospital.

### 3.2. Criteria and Experience of ED Clinician

Streaming was performed by the clinicians in conjunction with triage to UCC, following vital signs measure and clinical evaluation, based on CTAS scores and streaming/direction criteria (Table 1). Ideally, ED healthcare providers should have the skill, knowledge, and critical thinking to meet basic criteria to cover critical areas such as triage. Although requirements vary from hospital to hospital, not only they all agree that ED experience should not be less than 6 to 12 months for doctors and 2 years for nurses but also that they should be a basic life support (BLS) providers. For instance, advanced cardiovascular life support (ACLS) or advanced trauma life support (ATLS) are prerequisites in some critical areas such as ED.

In the context of the experience, highly experienced ED clinicians (physicians and nurses) made better and higher-quality streaming decisions either implementing a rapid front door streaming or a complex triage assessment inside the ED streaming using their intuitive, system-based and reflective judgements. Moreover, from the perspective of managers, they were considered to take a best decision about triage and direct patients with nonurgent problems to primary care services and to be more confident in communicating their decisions to patients and relatives, thus helping to improve patient flow and manage crowdedness.

“*Sometimes the right decisions are not carried out due to some medico legal issues against the doctors, their consequences as well as the recommendations of the higher authority*” (Triage physician at Ta hospital).

### 3.3. Streaming Decisions and Outcomes

Making decisions related to streaming practice based on “*System-aided judgement*,” means that the use of policies and procedures, clinical guidelines, computerised decision analysis systems to guide clinical judgement, and decision making. On the other hand, “*Reflective judgement*” applied through unformal way by some practitioners, special at peak time. It depends on healthcare providers' previous experience.

At Ma hospital, all patients under criteria for ESI-4 (nontrauma) and ESI-5 were selected for referred to UCC. Therefore, all stable ambulatory wheelchair patients presenting to emergency department will be screened and assessed on arrival, initially at visual triage by visual triage nurses in walking gate mainly for respiratory cases. At registration counter, the patient or relative will open digital ED number by clerk and give him/her printed barcode and then would be waiting to get triaged by triage nurse. However, the waiting time and reasons are explained to patients and relatives, usually it is around 20–30 minutes from registration.

As per the system, in hospital (Je), no patient who arrives at the emergency door is directed to Primary Health Centre (PHC). All cases are received/accepted and necessary service is provided either in the emergency department (ED) or Urgent Care Centre (UCC), concurrently. However, there are some excesses/transgressions from nursing at peak periods such as evenings, especially on weekends in directing cold and simple cases away into primary healthcare.

Moreover, the patients group list of ESI level-5, where no any resources are needed such as labs, radiology, and IV or IM medication. For example, old trauma, simple (abdomen, ear, eye, or lower back) pain, and healthy 52 years old who ran out of his blood pressure medication. On the other hand, the streaming decision to PHC may be influenced by patient's behaviour, age, language, and shortage of nurses. Consequently, streaming was unpreferable to some physicians, especially non-Saudi due to negative consequences of medic-legal issues (patient and family complaints) such as travel ban in Ta hospital. For instance, doctor-based triage was usually expected to face challenges, obstacles, and resistance from some patients/relatives, as well as nurse-based triage such as old age and less-educated people.

“*In real life situation, the decision to streaming/directing patients are objective more than subjective, Senior nurses where more competent and confident to control the overlap and crowded*” (Chief Director of ED at Hospital Ma).

Lastly, commitment to the streaming protocols and patient criteria varies from site to site and from streamers to another's. Although it is hard to commit in some situations such as (hysterical) patient beliefs and behaviour, some physicians prefer to escalate streaming decision to the higher authorities (duty manager) as avoidance and protection of negative consequences.

## 4. Discussion

The goal of this study was to explore the pathways (process) for redirecting patients from emergency department (ED) to Primary Health Centre (PHC) and to identify the streaming criteria and guidance. The results of this study found that the three study sites use two different types of triage scales, two of whom used the same scale CTAS. Even if the third hospital using the ESI scale, the two types have the same levels of categories. As a result, both types were streamed level-5 cases and some of level 4. In accordance with our results, a study conducted in the KSA revealed that all CTAS-5 cases can safely be triaged/streamed to PHC, as well as CTAS-4 can be triaged to either the PHC or an emergency centre, for example (CCU) [19].

The streaming is the process of allocation and redirecting of patients to most appropriate areas throughout the most convenient clinical pathways. However, the purpose of this streaming is to determine the best area for patients, from the first contact at the ED upon the patient arrival, either away (off-site) or (on-site) from emergency department by explained and trained clinicians (doctor/nurse) after initial assessment, this include brief history and basic vital signs (blood pressure, pulse, temperature, and oxygen situation). The results of this study explored that all CTAS-5 were streamed away either (off-site) or (on-site) from emergency department, which supported a previous study conducted in England and Wales found that there are the following three common emergency department pathways: front door streaming, streaming inside the emergency department, and primary care staff selecting patients [11, 20]. Moreover, streaming must be performed within 10–15 minutes as soon as possible, to ensure the quality of care provided to patients to meet their needs. In short, the total patients ranging from 50 to 60 per shift, which mean 15–20 case per hour and the most common cases were old trauma, flank pain, animal bites, ear pain, skin allergy without systemic symptoms, chronic back pain, chronic headache, and simple cut wounds.

In fact, the number of level-5 (nonurgent) cases flowing into the ED in the evening on the weekend ranged from 25 to 30 an hour, 10 to 15 of those streamed into the Urgent Care Centre (UCC), often about a third to half of cases. These findings are consistent with a study conducted in the KSA revealed that about (42.3%) of the emergency department patients were Class V (nonurgent), based on the CTAS classifications [9]. Totally, 200 to 240 cases are being sorting/triaged in the evening during the weekend and the average was about 27 cases per hour. However, it is likely that the reason for the high number and percentage is due to lack of public awareness of the existence of Urgent Care Centres and the services provided and their operating hours. In addition to the geographical location of the hospital and accessibility to the emergency departments, especially for visitors and pilgrims/Umrah performers during the seasons of religious rituals. One study reported that as contributing factor to inappropriate streaming [12]. Eventually, this number decreased by 10–15% in the morning period, about 13–19 cases per hour. At Ta Hospital, only 1–4 cases were streamed to UCC.

Furthermore, the primary care streaming pathways are designed to stream nonurgent patients away from ED to appropriate Primary Health Centre or Urgent Care Centre (PHC/UCC). Usually, the level-5 cases are the most common streaming based on the hospital policies and systems. At Ja hospital, on arrival to the emergency department entrance patient will be visually assessed immediately by specifically trained and experienced registered nurse within 2-3 minutes. Physician and nurse in triage area will assess the patient to determine patient's CTAS level. All patients triaged must be registered in electronic healthcare centre with vital signs in UC according to pathway. However, All CTAS-5 will be referred to the urgent care clinic by filling the urgent care referral form and should be registered electronically in the system. Rapid reassessment and retriage of CTAS-5 patients will be done once patient arrived at urgent care centre by the physician. In case patient needs further workup from lab investigation and radiological investigation he may contact ED specialist/consultant by hospital phone in the urgent care clinic. Overall, most cases only required oral medication, prescription will be given and discharge from urgent care clinic. On the other hand, cases that required admission, review by subspecialty, further workup which is outside the scope of service for urgent care or if any patient gets critical in urgent care clinic will be immediately triaged up and will be transferred to main ED department accompanied with physician, nurse, and paramedic to help in transport. At on-site model of care, the following services are provided in the Urgent Care Clinic (UCC):

1. Treat and discharge
2. Intramuscular injection
3. i-STAT laboratory test
4. Intravenous medication and fluids
5. Nebulisation
6. X-Ray investigation

On the other hand, the level of clinicians' awareness about the streaming process is critical for which patients and how they are selected? In addition, the usual time assessment elements as well as the factor that may influence the streaming? Study highlights many factors include Human factors such as complaint details, lack of knowledge and communication skills, patient behaviour and attitude, language barrier, and clinicians shortage and medic-legal issues such as complaints against the clinicians. This is consistent with a recent study of factors influencing streaming to general practitioners in emergency departments which highlighted success factors as well as issues that may contribute to poor streaming practice [5, 21]. Moreover, at Ma hospital, some especial factors such as accommodation and transportation cost for Umrah and visitor patients should not be ignored.

Indeed, factors explored are more likely among less experienced (junior), while it is less likely among senior experienced clinicians. As a result, previous studies have confirmed that experienced nurses, good teamwork, and strategic and operational management are key factors for primary care flow effectiveness [10, 22]. According to a recent study conducted in Saudi Arabia, nurses' knowledge regarding to triage has a positive relationship with practice [23]. Of note, noncompliance with the standards required for triage clinicians may underestimate or overestimate the score. However, in Ja and Ta Hospital (physicians-based triage) they must have 6 to 12 months experience in ED, while in Ma Hospital (nurse-based triage) they must have at least 2-year experience in ED. This result supported the findings of a previous study that found that there is a significant connection between nurses' triage knowledge and their years of experience in nursing [24]. On the other hand, digital triage system has been challenging at Ta Hospital and opportunity to change the triage system at Ma Hospital as they are planning. Therefore, the applicability of digital care to help streaming and triage patients focused on the skill and experience of clinicians in using the electronic triage system. Electronic triage system was found in a study conducted in Saudi Arabia as a way to enhance the health services provided to patients in EDs [25]. In spite of, it is one of the most effective ways to ensure appropriate and safe streaming decision, but measuring this effectiveness not reported nor recorded.

Finally, to overcome the factor of shortage of clinicians, we recommend that male and female triage be merged/consolidated into one triage area, in Ja Hospital.

### 4.1. Implications for Policy and Practice

To sum up with implications for stream and triage practice, we rely on hospital policies and procedures to weight decisions and outcomes. However, instead of following a one-way decision approach, we recommend collaboration approach between the ED clinicians (physicians/nurses) and patients to control the factors and decisions that shape their health lives, known as patient empowerment and engagement.

To support this recommendation in order to improve the quality of healthcare services and patient experience, we suggest the following strategies based on Saudi Patient Safety Centre (SPSC):

- Engaging beneficiaries/patients in decision making
- Empowering beneficiaries/patients in evaluating healthcare services
- Implementing of patient-centre care (PCC) concept
- Allowing patient to provide feedback, suggestion, and patient perception about the care they receive

From the nursing experience side, junior nurses must be engaged in various real emergency situations to arm them with knowledge/skills they need to make a triage decision. Furthermore, coaching with senior nurse will empower their streaming decision. Making the right decision for triaging patients will also help to reduce the overcrowding in EDs which in turns makes healthcare providers to provide the best quality of care for each patient needs to get treated in EDs within a specific period of time. Healthcare providers in EDs need to be aware of referring nonurgent patients to PHC centres is an essential step to minimise overcrowding in EDs and reduce the risk of the accessibility and quality of care for patients who are urgent.

### 4.2. Future Research

Further research that takes account of the effectiveness of the streaming paths is required to measure the patient safety and experience, quality of outcomes, and patient satisfaction. Moreover, streaming processes also need to be reevaluated to assess the patients accessing (off-site) appropriate services (especially Ummrah and visitors). We set a clear list of streams paths and operated PHC that is available to streamer, to achieve the four patient rights to health (availability, accessibility, acceptability, and quality) will be useful to meet our recommendations (Table 2). Future research could also focus on the extent of adherence to streaming protocols and guidance, as well as basic criteria for both the ED clinicians (physicians/nurses) and patients to ensure the effective streaming. We established clear protocols and software to assist streamer in determining the most appropriate area through the systematic streaming (digital) should be the priority of the future studies. More studies are needed regarding to the effects of overcrowding in EDs on providing available, accessible, acceptable, and best quality of care for each patient visiting EDs.

### 4.3. Limitations

There are some limitations for this study. One of the most challenging flows is meeting the standard criteria for the clinicians (physicians and nurses) who cover the triage area. In one emergency department, there was a mix of skills for the nursing staff (senior with junior nurse or senior with internship students) due to a shortage. Moreover, in two other “physician-based triage” burnouts was the most common cause of shortage, which could have a negative effect on streaming efficacy.

Unfortunately, this study included only three sites located in the Western Region of Saudi which could be affect the generalizability of the study results to be applied to others emergency departments (EDs) in the rest of the Kingdom due to geographical and population differences.

## 5. Conclusion

Through in-depth observation and unstructured interviews, it was observed/explored two pathways used to direct patients to primary healthcare, including the front door flow and the ED flow. This study explores lack of public awareness and knowledge in the role of streaming pathways. In general, the term “streaming” was not as widely known among ED clinicians, as was the term “triage.” However, streaming was performed as an evidence-based practice and clinicians routinely acted to direct patients based on hospital policies. Although, in Ja Hospital, some nurses hack the system to manage the flow of patients based on their intuition. In contrast, the nurses in the Ma hospital emphasised the importance of experience and confidence in streaming improvement.

## Figures and Tables

**Table 1:** Essential elements of the right to health.

Element	Definition
Availability	“Health resources must be available in sufficient quantities within the country to manage the population's needs, including trained personnel, healthcare facilities, and essential medicines”

Accessibility	“Health facilities, goods and services must be distributed in such a way as to be accessible to everyone without discrimination. Special consideration should be given to vulnerable populations, underserved geographic regions, and affordability”

Acceptability	“Health facilities and services should be respectful of medical ethics and culturally appropriate to the local context”

Quality	“Health facilities, goods, and services must be scientifically and medically appropriate and of good quality”

**Table 2:** Allocation and redirection methods.

Triage	“A clinical activity to sort patients by acuity so that those with the greater need are seen first”

Streaming	“An operational activity to assess whether low acuity patients are suitable to be seen by an appropriate non-ED clinician”

Simple assessment	“A brief “hands-off” assessment (i.e., no formal clinical assessment) that enables patients to be flowed to a suitable treating clinician”

Redirection	“Patients are sent to a care provider at another geographical site”

## Data Availability

Access to data is restricted due to ethical concerns and privacy of participants' information.
